# Dislocation of a WEB Device into the Middle Cerebral Artery: Removal with the Alligator Retrieval Device

**DOI:** 10.1007/s00062-018-0685-1

**Published:** 2018-05-11

**Authors:** I. König, A. Weber, W. Weber, S. Fischer

**Affiliations:** Universitätsklinik, Knappschaftskrankenhaus Bochum-Langendreer, Institut für Diagnostische und Interventionelle Radiologie, Neuroradiologie, Nuklearmedizin, Ruhr-Universität Bochum, In der Schornau 23–25, 44892 Bochum, Germany

## Introduction

Intra-aneurysmatic flow diverters offer an endovascular treatment option for broad-based intracranial aneurysms without the need for neck bridging devices (e. g., self-expandable stent, remodeling balloon) placed in the parent artery. Experience with the Woven EndoBridge (WEB) device (MicroVention, Tustin, CA, USA), as the only established intra-aneurysmatic flow diverter, is rapidly growing with promising angiographic and clinical results over the past years [1, 2].

Coil migration is a well-known complication in the endovascular treatment of intracranial aneurysms treated by standard coiling, which occurs in up to 6% of the cases. Several endovascular rescue strategies address the problem of partly or totally dislocated platinum coils ranging from oral antiplatelet therapy to dedicated devices especially designed for the retrieval of dislocated coils [3–5].

Here, we describe a case of a secondarily displaced WEB device during the treatment of an internal carotid artery aneurysm followed by successful withdrawal from the middle cerebral artery with an Alligator retrieval device (Medtronic, Dublin, Ireland).

## Case Report

**Fig. 1:**
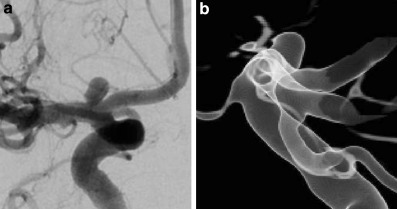
**a** Incidental aneurysm of the right internal carotid artery bifurcation, anterior-posterior view, **b** aneurysm morphology in 3D angiography

**Fig. 2:**
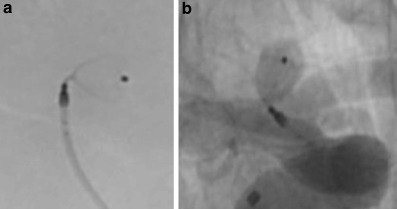
**a** Fluoroscopy after positioning of the WEB SLS 4 mm device documents the very tight angulation between the aneurysm and the internal carotid artery, lateral view, **b** appropriate position of the WEB device inside the aneurysm without any compromise of the parent artery, anterior-posterior view

A 64-year-old female patient was referred to our hospital with an aneurysm of the right internal carotid artery bifurcation. The aneurysm was diagnosed by magnetic resonance angiography (MRA) performed at the referring hospital after an episode of impaired vision. Using a diagnostic digital subtraction angiography (DSA) including a rotational 3D angiography the aneurysm morphology could be visualized in detail. Additional aneurysms were ruled out. The average aneurysm diameter was 2.9 mm with a maximum diameter of 3.1 mm measured in the lateral projection. The maximum aneurysm height was 3.2 mm (Fig. 1). The case was discussed in an interdisciplinary neurovascular board resulting in the recommendation for an endovascular treatment. After a comprehensive explanation of the risks and benefits, the patient decided for the suggested treatment strategy. The broad based longish morphology made this aneurysm suitable for treatment with a WEB device, although the angulation between the aneurysm and the carotid artery was very tight with a rostrally positioned inclination of the aneurysm (Fig. 2a).

**Fig. 3:**
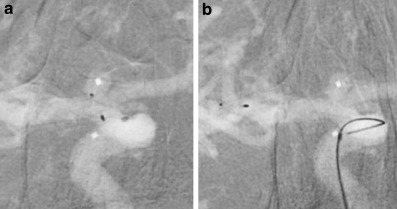
**a** Dislocation of the Web device after uneventful detachment during withdrawal of the microcatheter, anterior-posterior view, **b** secondary dislocation of the WEB device into the bifurcation of the middle cerebral artery, anterior-posterior view

According to our institutional standard the patient was placed on dual antiplatelet therapy 5 days prior to the procedure in order to obtain a bail out option including the placement of a stent. The procedure was carried out with the patient under general anesthesia. A coaxial guiding catheter combination (Neuron™ MAX 6F, Penumbra, Alameda, CA, USA) and Navien™ 072 (Medtronic, Irvine CA, USA) were positioned in the cervical segment of the right internal carotid artery. Size selection of the WEB device resulted from exact calibrated measurements of the aneurysm in two orthogonal projections based on a 3D rotational angiographic dataset according to the established standards described in the literature [2]. A WEB SLS device, the more spherical version of the WEB with a 4 mm width, was chosen in the particular case. A VIA 17 microcatheter (MicroVention) was placed in the center of the aneurysm followed by the implantation of the WEB device. Once the WEB was completely unsheathed from the microcatheter an angiographic run documented the appropriate position of the device inside the aneurysm without any compromise of the parent artery (Fig. 2b). A further angiographic run 10 min later confirmed the stable position of the device. The device was than electrothermally detached with the distal marker of the microcatheter placed towards the detachment zone of the WEB. The detachment from the insertion wire was without problems. The cautious withdrawal of the microcatheter resulted in a dislocation of the WEB device outside the aneurysm into the middle cerebral artery. The next angiographic run documented a further dislocation of the device that was now locked inside the bifurcation of the middle cerebral artery (Fig. 3).

**Fig. 4:**
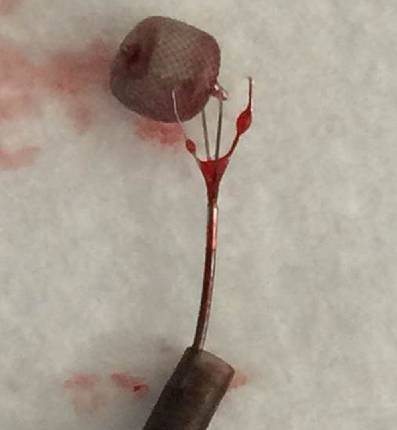
The WEB device trapped between the jaws of the Alligator retrieval device

Now a Rapid Transit microcatheter (Codman, Norderstedt, Germany) was advanced towards the dislocated WEB device with a Traxcess EX microwire (Microvention). The microwire was pulled back and the Alligator retrieval device was inserted into the microcatheter and pushed forward. Once the closed jaws of the device reached the distal marker of the microcatheter both the microcatheter and the device were pushed towards the struts of the WEB device. Now the Alligator retrieval device was slightly advanced and the microcatheter was held in place, which resulted in an opening of the four jaws of the Alligator device at the level of the dislocated WEB. The microcatheter was then slightly advanced under permanent distally directed tension of the Alligator retrieving device in order to close the jaws. At this point the ensemble of the microcatheter with the Alligator was gently pulled back with the WEB device trapped between the jaws (Fig. 4).

A final angiographic run proved an unchanged situation especially without suspicion of a dissection followed by the described maneuver. The procedure was finished without a final treatment of the aneurysm and 5 days later the aneurysm was occluded with coils using the remodeling technique.

## Discussion

The endovascular treatment options for broad-based intracranial aneurysms are nowadays various ranging from stent-assisted coiling to extra-aneurysmal or intra-aneurysmal flow diversion. The identification of the optimal treatment strategy in a particular case can be challenging. Growing experience with a new technique, and especially its potential limitations, helps to identify the appropriate indications.

With respect to the literature published to date a complete dislocation of a WEB device outside an aneurysm is a unique complication of this technique. The reason for this complication finally remains unclear but some possible explanations are hypothesized as follows: we followed the recommendation to oversize the width of the WEB device with a compensatory undersizing of its height in order to clamp the device inside the aneurysm according to the so-called +1/−1 rule [2, 6]. These above mentioned diameters would require a 3.5 × 2 mm WEB SL device according to the current selection guide of the manufacturer, indicating that the +1/−1 rule applies more for the mid-sized aneurysms with diameters from 5–7 mm; however, the interventionalist decided to use the slightly oversized 4 mm SLS device with respect to the spherical shape of the aneurysm in order to achieve a correct reconstruction of the neck area. The clear oversizing of the device might be one reason for the dislocation especially in combination with the complex anatomy in this case and the detachment maneuver as described.

As documented in Fig. 2a the angulation between the longitudinal axis of the aneurysm and the terminal carotid artery was almost 90° in the lateral view. Many studies proved a high efficacy of the WEB device especially in complex anatomies of bifurcation aneurysms with tight angulations of efferent branches that are therefore difficult to treat by stent-assisted techniques [1, 2, 6–8]. Nevertheless, a tight angulation between the parent artery and the aneurysm might indicate a more challenging procedure regarding the positioning as well as the final detachment of the WEB device.

The distal marker of the VIA microcatheter was advanced towards the WEB device in order to cover the detachment zone and promote the detachment process, as recommended by the manufacturer. Regarding this advice, awareness of the peculiar position of the distal marker on the VIA microcatheter is important since it does not indicate the exact end of the catheter but is placed 1 mm proximal to the definite end of the catheter. These technical features might explain the dislocation of the WEB in our case. The close contact of the proximal marker of the WEB with the distal end of the VIA microcatheter might in combination with the tight angle between the WEB and the microcatheter have caused an interlocking of both followed by a mobilization of the WEB device during the withdrawal of the microcatheter.

Several endovascular devices address the problem of dislocated material ranging from snares to stent retrievers [9–11]. The idea to remove the expanded WEB with a snare or a stent retriever did not appear to be promising to us. Probably the secondary displacement of the WEB into the bifurcation of the middle cerebral artery facilitated the removal since the fixed WEB provided an abutment for the Alligator device.

## Conclusion

Detachment of the WEB device and withdrawal of the microcatheter should be performed with caution especially in complex anatomies with tight angles between the aneurysm and the parent artery. The position of the distal marker on the VIA microcatheter might be misleading.
